# Loneliness, Depressive Mood and Cyberbullying Victimization in Adolescent Victims of Cyber Dating Violence

**DOI:** 10.3390/ijerph17124269

**Published:** 2020-06-15

**Authors:** María-Jesús Cava, Inés Tomás, Sofía Buelga, Laura Carrascosa

**Affiliations:** 1 Faculty of Psychology, Department of Social Psychology, University of Valencia, Avda. Blasco Ibáñez, 21, 46010 Valencia, Spain; Sofia.Buelga@uv.es; 2 Faculty of Psychology, Department of Methodology of the Behavioural Sciences, University of Valencia, Avda. Blasco Ibáñez, 21, 46010 Valencia, Spain; Ines.Tomas@uv.es; 3 Department of Social Sciences, Valencian International University-VIU. Calle Pintor Sorolla, 21, 46002 Valencia, Spain; laura.carrascosa@campusviu.es

**Keywords:** cyber dating violence, victimization, cyberbullying, loneliness, depressive mood, gender analysis, adolescents

## Abstract

Currently, cyber dating violence (CDV) is a serious health problem among adolescents due to their frequent use of communication technologies in their romantic relationships including the use of these technologies to perpetrate dating violence. However, research on this topic is recent and more studies about victims’ psychosocial adjustment are needed. The objectives of this study were to analyze the prevalence of CDV victimization according to frequency (occasional and frequent) and type (cyber control and cyber-aggression) and to explore their relations with loneliness, depressive mood and cyberbullying victimization. A total of 604 adolescents (*M* age = 14.32, *SD* = 1.67) who had a dating relationship at the time or in the past 12 months, participated in this study. The results showed a higher prevalence for cyber-control than cyber-aggression victimization, and positive correlations of CDV victimization with depressive mood and cyberbullying victimization in boys and girls. Positive correlations with loneliness were also observed for girls. The average effect size of the aforementioned correlations was large for girls and medium for boys. Both boys and girls who were frequent victims of CDV also suffered more cyberbullying by peers than those who were never, and occasionally, cyber victimized by their partners. Girls who were frequent victims of CDV also reported higher scores for loneliness and depressive mood, with a small average effect size. All these results highlight close relations between cyberbullying and CDV in adolescents, being necessary to pay greater attention to possible experiences of poly-victimization, and a worse psychosocial adjustment in frequently victimized girls than boys. These findings may be useful for developing more effective intervention programs.

## 1. Introduction

Cyber dating violence (CDV) is an increasing public health problem because adolescents constantly use information and communication technologies (ICTs) in their daily lives, including their interpersonal relationships [1,2,3]. Currently, smartphones are an essential element in the social life of adolescents and young people worldwide, which means that the virtual world is increasingly important in their lives [2]. Adolescents and young people use communication technologies to initiate, maintain and end romantic relationships [4,5,6]. These communication technologies facilitate affective links with romantic partners, but can also be used to perpetrate dating violence [6,7,8,9]. CDV is defined as the control, threat, harassment, stalking and abuse of current or former dating partners via technology and social media [5,9,10,11,12,13,14,15]. This cyber violence includes many forms of abusive behaviors, such as daily control through social networks, sending offensive or humiliating comments to dating partners, sending messages with different threats or posting photos with the intention to humiliate dating partners or harm them [4,16,17,18,19]. These different behaviors have been grouped according to two types: cyber-control and cyber-aggression [11,20,21,22]. Cyber-control includes different forms of controlling partners by technology to monitor their social relationships, what they do at any time, where they are and with whom. Cyber-aggression includes behaviors that involve harming partners through direct attacks, e.g., sending threats and insults or disseminating private information on social networks [11,12,21,22,23,24].

Previous studies have analyzed CDV prevalence among adolescents, and have shown worrying percentages of adolescents involved in this cyber violence [19,25,26]. Nevertheless, these studies offer very different percentages of adolescents suffering CDV victimization [5]. Zweig et al. [19] reported 26% for adolescent CDV victims, while Marganski and Melander [27] reported 73%. In their review, Stonard et al. [26] indicated that CDV victimization rates ranged from 12% to 56%. This wide variability encountered in studies may be due to differences in the measurement instrument employed and the type of measured behaviors [5]. In previous studies that have differentiated between cyber-control and cyber-aggression behaviors, a higher prevalence of cyber-control was observed [7,21,25,28]. This higher prevalence of cyber-control behaviors has been related to adolescents being less aware of these behaviors as a form of cyber abuse given their belief in myths of romantic love that make them even consider such behaviors to be signs of love, e.g., linking jealousy and control with love [11,29]. Cyber-control behaviors can increase in frequency and intensity over time, and may be a first step to more serious online and offline teen dating violence later on [6]. However, these behaviors may only be occasional and linked with adolescents’ lack of previous experience in romantic relationships [30]. Adolescents’ beliefs in myths of romantic love (e.g., taking jealousy to be a sign of love), along with them lacking previous experience in handling conflicts in their first romantic relationships, might explain their occasional involvement in cyber-control behaviors [31]. In their first romantic relationships, adolescents might not perceive some occasional cyber-control behaviors as CDV, so they might have a less negative impact on their well-being. In one previous study focused on offline dating violence victimization, with a sample of 672 adolescents, significant differences in psychosocial adjustment between adolescents occasionally and frequently victimized by their partners were observed, but there were no significant differences between occasionally and never victimized adolescents [31]. In this study, the adolescents who were frequently victimized reported lower family self-concept, and had more communication problems with the mother, more depressive mood, more feelings of loneliness and less life satisfaction than those occasionally victimized [31]. Although the effect sizes of the differences reported in this study were small (with eta-square values ranging from 0.011 to 0.045), small effects can matter in the long run [32]. As pointed out by Funder and Ozer ([32], p. 161), “a psychological process that affects the behavior of a single individual repeatedly over time…., can have hugely important implications”. In a similar way, adolescents’ psychosocial adjustment could also differ according to the frequency and type of CDV (cyber-control or cyber-aggression) that they are suffering. Adolescents who experience occasional or frequent CDV may differ widely in their psychosocial adjustment. These possible differences should be examined in more depth.

In addition, relations between CDV victimization and psychosocial adjustment might be different in adolescent boys and girls. Although some previous studies have not found any gender differences in CDV victimization prevalence [15,33], other studies have reported a higher prevalence for girls [9,19,34,35] and others for boys [16]. In this regard, Caridade et al. [5] reported in their review that results about gender differences were inconclusive. Nevertheless, a greater knowledge on gender differences in CDV victimization needs to analyze not only the prevalence, but also the frequency and type of CDV suffered by boys and girls and possible gender differences in the relationships with the adolescents’ psychosocial adjustment.

### 1.1. Cyber Dating Violence Victimization, Depressive Mood and Loneliness

CDV victimization has a very negative impact on adolescents’ psychosocial adjustment. This cyber violence can occur constantly, and the humiliating and threatening messages by partners can take place 24/7, without victims being able to stop attacks. Additionally, a victim’s humiliating photos and information can quickly be spread to a wide audience. All these characteristics increase victims’ feelings of helplessness and hopelessness, who then feel unable to avoid the cyber aggression [6,9,14,19,21,28,36,37,38,39]. The absence of geographical and temporary barriers in the virtual world mean victims might suffer CDV at any time and from anywhere, which is an important difference compared to offline dating violence. CDV is considered qualitatively different from offline dating violence and may have even more negative outcomes than offline victimization [6,9,14,15,19,40]. Previous studies have linked CDV victimization with depressive symptoms [9,28], anxiety [28], suicide risk [7], emotional/psychological distress and low self-esteem [15,36]. However, some researchers have suggested that adolescents’ perception of some abusive behaviors as normal behaviors in a romantic relationship can minimize these negative outcomes [41,42]. Cyber-control victimization, especially when it is occasional, may not be perceived as CDV victimization by adolescents and could be less related to problems in adolescents’ psychosocial adjustment. Therefore, more studies that analyze the relations between CDV victimization and indicators of psychosocial adjustment, such as the depressive mood, that take into account the suffered cybervictimization type (cyber-control or cyber-aggression) and frequency (occasional or frequent) are needed.

Another important indicator of psychosocial adjustment that could be related to suffering CDV is loneliness. Feelings of loneliness have been related to offline dating violence victimization [43], and social isolation is often desired by aggressors who attempt to control their victims [44]. Adolescent CDV victims can feel loneliness if partners control their online social relationships and with whom they may maintain online friendships. Moreover, if their personal image on social networks is damaged through spreading false rumors and humiliating information about them in social media, they can feel especially isolated. During adolescence development, identity is consolidated and adolescents are particularly worried about their public self-image and possible peer rejection [45]. If demeaning personal information and humiliating photos about them are disseminated to a large audience, their psychological distress and feelings of loneliness can greatly intensify. Relations between CDV and loneliness have not been previously analyzed, but these links have been observed in offline dating violence [31,43], and specific CDV characteristics can make these links even stronger. Furthermore, there are correlations between CDV and offline dating violence [7,11,16,27,39,46], and many adolescents can suffer both online and offline dating violence. Nevertheless, the links between feelings of loneliness and CDV victimization can differ according to the frequency of victimization suffered. In offline dating violence, frequently victimized adolescents display more feelings of loneliness and assess their social network more negatively than those not victimized or occasionally victimized [31]. Along the same lines, the frequency and type of CDV suffered by adolescents should be taken into account when analyzing their relations with adolescents’ feelings of loneliness.

### 1.2. Cyber Dating Violence Victimization and Cyberbullying Victimization

The current use of ICTs by people in their daily lives also increases cyberbullying in adolescents and young adults [2]. Cyberbullying is defined as an intentional, aggressive and repetitive behavior, where a person or group uses electronic devices to bully a person who cannot defend themselves [2,47,48,49]. There are direct and indirect forms of cyberbullying [2,8,50]. Direct forms of cyberbullying include acts in which a victim is directly attacked by one or several perpetrators through, for example, threatening text messages or social network site posts, or by removing him/her from groups to isolate them. Indirect forms of cyberbullying involve acts that cause the victim indirect harm, such as creating a false profile, hacking their personal account, or sharing entrusted personal information with others (e.g., posting intimate text messages or images in a public online area). Similar to CDV, direct and indirect cyberbullying are characterized by the absence of any geographical or temporal limitations, which makes it very difficult for victims to escape abuse [8,50]. Cyberbullying and CDV share common features, such as the online permanence of victims’ humiliating information, the possible public nature of aggression, and the ease with which to retrieve and share images, rumors and demeaning information through social media in order to harm the victim [8,13,14,17,32,41].

Links between cyberbullying and CDV have been previously reported [5,21], and possible common risk factors have been suggested [8]. Nevertheless, research on the relations between them is still scarce. One interesting question to examine is the possible connections between being victimized by both cyberbullying and CDV. Adolescent victims in one interpersonal context are more probable to also be victimized in other interpersonal contexts or through other forms of victimization, and they can suffer situations of poly-victimization [42,43,51,52]. The connection between teen dating violence victimization and peer victimization [39,43,53,54,55,56,57], and the links between offline and online dating violence victimization [11,22,27,39], have been pointed out. Thus it is likely that both forms of cybervictimization are closely linked. A more in-depth analysis of these relations, by taking into account the type and frequency of CDV victimization and possible gender differences, could be very useful for detecting adolescents in more vulnerable situations and preventing possible future victimizations.

### 1.3. The Current Study

Taking into account previous research and the need for more in-depth analysis of the relations of CDV victimization with cyberbullying victimization, loneliness and depressive mood in adolescents, three objectives were proposed in this study. The first objective was to analyze the prevalence of CDV victimization in adolescent boys and girls by taking into account the frequency (never, occasional and frequent) and type (cyber-control and cyber-aggression) of CDV suffered by adolescents. The second objective was to analyze the correlations between CDV victimization (cyber-control and cyber-aggression), feelings of loneliness, depressive mood and cyberbullying victimization (direct and indirect). Finally, the third objective was to analyze any possible differences in loneliness, depressive mood and cyberbullying victimization in adolescents with a different type (cyber-control and cyber-aggression) and frequency (never, occasional and frequent) of CDV victimization. Regarding these objectives, we proposed three hypotheses that were justified in the empirical results reported in the previous literature.

**Hypothesis** **1.** *The prevalence of cyber-control victimization in adolescent couples will be higher than cyber-aggression victimization for both boys and girls. More specifically, we hypothesize a prevalence higher than 30% for cyber-control victimization and higher than 10% for cyber-aggression victimization. Borrajo et al. [21] observed a prevalence of 75% for cyber-control victimization and 14% for cyber-aggression victimization with a sample of 788 young adults from 18 to 30 years old, and Gracia-Leiva et al. [7] indicated a prevalence of 66.7% for cyber-control victimization and 33.3% for cyber-aggression victimization with adolescent females aged 13–28 years. However, we hypothesize a prevalence slightly lower for CDV victimization among adolescents, in line with a previous study that reported a prevalence of 55.9% for cyber-control perpetration and 10.1% for cyber-aggression perpetration among adolescents aged 12–18 years old [29]. Moreover, taking into account that prevalence is higher for occasional than frequent offline dating violence victimization in adolescent couples [31], we anticipate a higher prevalence for occasional than frequent CDV victimization.*

**Hypothesis** **2.** *CDV victimization (cyber-control and cyber-aggression) will be positively correlated with loneliness, depressive mood and cyberbullying victimization (direct and indirect) in both boys and girls. In a previous study carried out by Borrajo and Gámez-Guadix [28] with a sample of 782 youths, depressive mood was positively correlated with cyber-control victimization (r = 0.27) and cyber-aggression victimization (r = 0.24); and another study by Borrajo et al. [21], with 788 young adults, showed positive correlations of cyberbullying victimization with cyber-control victimization (r = 0.18) and cyber-aggression victimization (r = 0.33). Correlations between CDV victimization and adolescents’ feelings of loneliness have not been analyzed previously, at least as far as we know. Nevertheless, positive correlations between offline dating violence victimization (relational, physical and verbal) and adolescents’ feelings of loneliness were reported for both boys (r values ranging from 0.18 to 0.22) and girls (r values ranging from 0.16 to 0.18) in a previous study with a sample of 647 adolescents aged 12–17 years [43]. Based on this previous literature, we hypothesize positive correlations, with medium effect sizes, among all the analyzed variables for both boys and girls.*

**Hypothesis** **3.** *Adolescent boys and girls who frequently suffer CDV victimization (cyber-control or cyber-aggression) will report significantly higher scores for loneliness, depressive mood and cyberbullying victimization (direct and indirect) than adolescent boys and girls who have never suffered CDV victimization. Possible differences in loneliness, depressive mood and cyberbullying victimization according to the frequency and type of CDV victimization suffered by adolescents have not been previously analyzed. However, in a previous study with 672 adolescents aged 12–17 years [31], those who have frequently suffered offline dating violence showed more emotional loneliness and depressive mood than those never or occasionally victimized by their partners, with small effect sizes (emotional loneliness: η^2^ = 0.031, depressive mood: η^2^ = 0.045). Moreover, close relations between offline peer victimization and offline dating violence victimization (physical and verbal) have been observed [43], with medium and large effect sizes (r values ranging from 0.13 to 0.31). Based on these studies, we hypothesize small effect sizes for mean differences in loneliness and depressive mood among adolescents frequently, occasionally and never cyber victimized by their partners; and medium effect sizes for differences in cyberbullying victimization among these three groups of adolescent boys and girls.*

## 2. Materials and Methods

### 2.1. Participants

Our initial research sample comprised 1063 adolescent boys and girls, aged between 12 and 17 years (*M* age = 14.11; *SD* = 1.75). Regarding gender, 49.4% of these adolescents were boys (*M* age = 14.09; *SD* = 1.75) and 50.6% were girls (*M* age = 14.15; *SD* = 1.74). They all studied in three public compulsory secondary education schools in the Valencian region (east Spain). Most adolescents (70.2%) lived with both parents, 18.8% lived with their mothers, 2.5% lived with their fathers, 6.1% lived in shared custody situations, and 2.4% with other relatives. Of this initial sample, only the adolescents in a romantic relationship at the time, or in the past 12 months, were included in this study. These adolescents were asked to fill in scales about their romantic partner, by referring to the latest relationship they were in or had been in. They were asked to think about their last romantic relationship that lasted longer than a single date/a single encounter.

By considering the aforementioned inclusion criterion, the final study sample included 604 adolescents, 262 boys (43.4%) and 342 girls (56.6%), aged between 12 and 17 years. The mean age of boys (*M* = 14.27; *SD* = 1.69) and girls (*M* = 14.37; *SD* = 1.66) was similar. The highest percentages of adolescents were 13 (24.8%), 14 (19%), and 15 (18%) years old, with lower percentages for the 12- (13.4%), 16- (11.6%) and 17-year-olds (13.1%). In this final sample, 67.8% of adolescents lived with both parents, 21.9% only with their mothers, 2% only with their fathers, 5.3% in shared custody situations and 2% with other relatives. Most adolescents indicated having a heterosexual romantic relationship (95.5%), and only 4.5% admitted having a homosexual romantic relationship. Regarding the length of their romantic relationship, the majority of adolescents (52.9%) reported romantic relationships lasting between 1 and 6 months, with lower percentages of romantic relationships lasting less than 1 month (15.8%), between 6 months and 1 year (17.5%) and over 1 year (13.4%).

### 2.2. Instruments

Cyber-violence in adolescent couples scale [22]. This scale evaluates different forms of CDV perpetration and victimization in adolescent couples. The scale is composed of two sub-scales, cyber-violence perpetrated and cyber-victimization. In this study only the sub-scale of cyber-victimization was used. This sub-scale includes 10 items describing different aggressive and control behaviors that their romantic partner may have perpetrated against them through social media. These 10 items are integrated into two factors: Cyber-control (five items describing excessive control behaviors; e.g., “My partner doesn’t let me chat with some friends and if I do he/she get angry”) and Cyber-aggression (five items related to threats and insults through social media; e.g., “My partner has spread malicious rumors or lies about me though social networks”). Adolescents responded to these items using four options: 1 (never), 2 (seldom), 3 (sometimes) and 4 (often). The reliability (Cronbach’s alpha) of these two factors in this sample was 0.86 for cyber-control and 0.88 for cyber-aggression.

Loneliness Scale [58]. This scale includes two factors: Emotional loneliness, composed of 11 items about perceived emotional loneliness (e.g., “How often do you feel that you lack companionship?”) and subjective social network assessment, composed of nine items related to the subjective evaluation of the social support available in the person’s social network (e.g., “How often do you feel that there are people you can turn to?”). Adolescents responded to these items with four response options ranging from 1 (never) to 4 (always). The reliability (Cronbach’s alpha) of these two factors in this sample was 0.88 for emotional loneliness and 0.89 for subjective social network assessment.

Depressive mood scale [59]. This scale consists of seven items (e.g., “I had trouble keeping my mind on what I was doing”) that evaluate different characteristics of depressive symptomatology (loss of appetite, sleep problems, difficulties concentrating) and provide a general index of depressed mood. Adolescents responded to these items on a scale ranging from 1 (never) to 5 (always). Cronbach’s alpha of this scale in this sample was 0.91.

Cyber-bullying victimization scale [60]. This scale consists of 18 items that describe different experiences as victims of cyberbullying through mobile phones and the Internet. This scale includes two factors: Direct cybervictimization and indirect cybervictimization. Direct cybervictimization is composed of eight items related to cyber-aggressions directly oriented toward the victim, both verbal (e.g., “Someone insulted or ridiculed me in social networks or groups like WhatsApp to really hurt me”) and social (e.g., “Someone eliminated or blocked me from groups to leave me without any friends”). Indirect cybervictimization includes 10 items describing indirect cyber-aggressions, such as creating a false profile of the victim, identifying theft or hacking their personal account (e.g., “Someone logged into my profile or accounts and I could not avoid it”). Adolescents indicated how often they had suffered these experiences in the last 12 months: 1 (never), 2 (once or twice times), 3 (from 3 to 5 times), 4 (from 6 to 10 times), and 5 (more than 10 times). The reliability (Cronbach’s alpha) of these two factors in this study was 0.89 for direct cybervictimization and 0.91 for indirect cybervictimization.

### 2.3. Procedure

The initial contact made with secondary schools was by telephone. They were offered brief information about the research and a meeting with staff was requested to explain the research objectives in more detail. During this meeting with staff, the objectives were explained and their collaboration was requested. Adolescents’ families were informed about the research proposal by letter, which indicated that all the data would be confidentially used. After receiving this information, they were asked to give consent for their children to participate. Less than 1% of parents stated that they did not want their children to participate in this study. Adolescents anonymously and voluntarily filled out the scales during a regular class period (55 min). Trained researchers administered the instruments to the adolescents on one school day, and informed them that their participation in the study was voluntary and anonymous, their data were confidential and they could drop out of the study at any time. None refused to participate in this research. This study was approved by The Ethics Committee of the University of Valencia (Protocol Number: H1456762885511).

### 2.4. Data Analyses

First, CDV victimization (cyber-control and cyber-aggression) prevalence in adolescent boys and girls was calculated. In the prevalence analysis, we considered not only the type (cyber-control and cyber-aggression), but also the frequency (occasional and frequent), of CDV victimization suffered by adolescents. Following the criteria used in other previous studies [31,61], we considered frequent victimization when the scores in this variable exceeded the mean, plus one standard deviation. Thus, adolescents whose scores exceeded the mean score for cyber-control victimization by one standard deviation (score > 1.81; *M* = 1.29, *SD* = 0.52) were assigned to the group of “frequent cyber-control victimization”. Those adolescents who reported having never been victims of any behavior of cyber-control in their dating relationships were assigned to the group “never cyber-control victimization”. Finally, those adolescents who had suffered some cyber-control victimization behaviors, but whose scores did not exceed the mean score by one standard deviation, were assigned to the group “occasional cyber-control victimization”. Likewise, the adolescents whose scores exceeded the mean cyber-aggression victimization score by one standard deviation (score > 1.38; *M* = 1.08, *SD* = 0.30) were assigned to the group of “frequent cyber-aggression victimization”. The adolescents who reported having never been victims of any cyber-aggression behavior were assigned to the group “never cyber-aggression” and those who had suffered some cyber-aggression victimization behaviors, but their scores did not exceed the mean score by one standard deviation, were assigned to the group “occasional cyber-aggression”. We analyzed the prevalence of these different types and frequencies of CDV victimization separately for boys and girls.

Next, Pearson correlations among CDV victimization (cyber-control and cyber-aggression), loneliness (emotional loneliness and social network assessment), depressive mood and cyberbullying victimization (direct and indirect) were calculated for boys and girls. Possible gender differences in all these variables were analyzed by the Student’s t test for independent samples. The possible differences in loneliness (emotional loneliness and social network assessment), depressive mood and cyberbullying victimization (direct and indirect) between the adolescents suffering a different type and frequency of CDV victimization were analyzed by applying a multivariate analysis of variance (MANOVA). A first multivariate analysis of variance was performed to compare possible differences in loneliness, depressed mood and cyberbullying victimization according to the frequency of cyber-control suffered by adolescents (never, occasional and frequent). A second multivariate analysis compared possible differences in the same outcome variables according to the frequency of cyber-aggression suffered (never, occasional and frequent). These analyses were carried out separately for boys and girls and were all performed with the SPSS-26 statistical package. To interpret the effect sizes of the correlations among variables, we followed the guidelines proposed by Funder and Ozer [32] regarding Pearson’s *r*. Cohen’s *d* and eta-square values were used to interpret the effect sizes of mean differences estimated with the Student’s t test and MANOVA, respectively.

## 3. Results

### 3.1. Cyber Dating Violence Victimization Prevalence in Adolescents According to Type and Frequency

Regarding CDV victimization prevalence (Table 1), almost half the adolescents (44.1%) reported having suffered at least one cyber-control behavior by their partner. Of these adolescents, most had suffered occasional cyber-control victimization (30.5%) and 13.6% frequent cyber-control victimization. Therefore, one third of adolescents indicated that they had occasionally suffered cyber-control behaviors by their partners, and more than one in 10 had done so frequently. The distribution of boys and girls in the frequency-based groups of differing cyber-control victimizations showed no significant differences (χ^2^(2) = 5.530; *p* > 0.05). Cyber-aggression prevalence was significantly lower than cyber-control victimization (Z: −13.274; *p* < 0.01), but was also worrying. The results indicated that 11.6% of all adolescents had suffered at least one cyber-aggression behavior in their dating relationships. This cyber-aggression victimization was occasional for 4.1% of adolescents and frequent for 7.5% of them. The analysis of gender differences in adolescents’ distribution across these frequency groups showed significant differences between boys and girls (χ^2^(2) = 10.970; *p* < 0.01). A higher frequent cyber-aggression victimization prevalence was found for boys than for girls.

### 3.2. Means, Standard Deviations and Correlations among Variables

Table 2 shows the means, standard deviations and correlations among the analyzed variables in adolescent boys and girls. In girls, the results indicated significant correlations among all the variables. Both CDV victimization forms (cyber-control and cyber-aggression) were related positively to emotional loneliness, depressive mood and direct and indirect cyberbullying victimization, and negatively related to social network assessment. In boys, positive correlations were also observed among CDV victimization forms (cyber-control and cyber-aggression), depressive mood and direct and indirect cyberbullying victimization. However, in boys, cyber-control and cyber-aggression victimization were not significantly related to emotional loneliness. Regarding the mean gender differences, girls reported more feelings of emotional loneliness, depressive mood and direct cyberbullying victimization, and boys more cyber-aggression victimization in their dating relationships.

### 3.3. Loneliness, Depressive Mood and Cyberbullying Victimization in Adolescents According to Type and Frequency of Cyber Dating Violence Victimization

The results of analyzing differences in loneliness (emotional loneliness and social network assessment), depressive mood and cyberbullying victimization (direct and indirect) in adolescent boys and girls with distinct cyber-control victimization experiences (never, occasional and frequent) are shown in Table 3. Frequently victimized boys obtained significantly higher scores for depressive mood, direct cyberbullying victimization and indirect cyberbullying victimization than those never and occasionally victimized. However, no significant differences were found in any of these variables between never and occasionally victimized boys. No significant difference was observed in emotional loneliness and social network assessment in the frequently, occasionally and never victimized boys. In girls, the results revealed significant differences in all the analyzed variables according to their different cyber-control victimization experiences. The frequently victimized girls reported higher scores for emotional loneliness, depressive mood and direct and indirect cyberbullying victimization than the never victimized girls. Their scores were also higher in direct cyberbullying victimization than those occasionally victimized. The frequently victimized girls also indicated a more negative assessment of their social network than those never and occasionally victimized, with no significant differences in this variable between those never and occasionally victimized.

The results about cyber-aggression victimization also showed significant differences in more variables in girls than boys (see Table 4). In boys, significant differences only appeared between those frequently and never victimized in direct and indirect cyberbullying victimization. The boys who had frequently suffered cyber-control victimization in their dating relationships reported higher scores in direct and indirect cyberbullying victimization than those never victimized. There were no significant differences in either direct cyberbullying victimization between occasionally and frequently victimized or indirect bullying victimization between never and occasionally victimized. Nevertheless, in girls, significant differences were found in all the analyzed variables. The girls who reported having frequently suffered cyber-aggression victimization in their dating relationships obtained significantly higher scores for emotional loneliness, depressive mood and direct and indirect cyberbullying victimization than those never victimized. The frequently victimized girls also obtained significantly lower scores in the positive assessment of their social network. Significant differences also appeared in direct and indirect cyberbullying victimization between occasionally and never victimized girls. The scores in these two forms of cyberbullying victimization were higher in the occasionally victimized girls than those never victimized.

## 4. Discussion

A first objective of this study was to analyze CDV victimization prevalence in adolescent boys and girls by considering type and frequency of cybervictimization. The results of the present study confirmed a higher cyber-control victimization prevalence [7,21,25,28], with a similar prevalence in boys and girls. However, the percentage of adolescents who reported having suffered at least one cyber-control behavior by their partners (44.1%) was lower than the percentages obtained in previous studies with young couples and older adolescents: 66.7% of cyber-control victimization was observed by Gracia-Leiva et al. [7] with adolescents aged 13–28 years, and 75% by Borrajo et al. [21] with young people from 18 to 30 years old. The higher cyber-control victimization prevalence in older couples might be related to romantic relationships being longer in late adolescence and youth compared to early and middle adolescence. Romantic relationship length is a variable associated with greater cyber-control perpetration [41]. Regarding cyber-aggression victimization, the percentages of adolescents who reported having suffered at least one cyber-aggression by their partner (11.6%) were also lower than in previous studies with older couples: 33.3% observed by Gracia-Leiva et al. [7] and 14% by Borrajo et al. [21]. Although the longer length of romantic relationships during late adolescence and youth could also explain these higher percentages, other possible related variables should be explored in future studies.

Moreover, our results not only showed that cyber-control victimization prevalence was higher than cyber-aggression victimization, but most of the adolescents who had suffered this type of CDV reported occasional cyber-victimization (30.5%). Almost one in three adolescents reported suffering occasional cyber-control by their partner, while 13.6% reported situations of frequent cyber-control. These results confirm the initial hypothesis by showing a higher prevalence for occasional cyber-control victimization in adolescent couples, and could also indicate a certain normalization of such behaviors in these couples. Regarding cyber-aggression, the percentage of occasional victimization was similar and even lower (4.1%) than the percentage of frequent victimization (7.5%). In cyber-aggression, adolescent boys reported more frequent victimization than girls. Borrajo and Gámez-Guadix [28] also observed a higher cyber-aggression victimization prevalence in males than females with a sample of youths whose ages ranged from 18 to 30 years, although this study did not differentiate between occasional and frequent cybervictimization. These gender differences in cyber-aggression victimization need to be further analyzed in future research, by considering not only frequency, but also the motives of perpetrators and consequences for victims. For example, some characteristics of cyber-aggressions, such as the physical distance between the aggressor and victim that allow for avoidance of coping with the victim’s immediate reaction [6], could favor CDV perpetration by girls in response to previous offline and online dating violence victimization. All these questions need to be analyzed more broadly.

Regarding the correlations among CDV victimization (cyber-control and cyber-aggression) and adolescents’ psychosocial adjustment, the results showed some differences between adolescent boys and girls. Although positive correlations between depressive mood and CDV (cyber-control and cyber-aggression) victimization were confirmed in both boys and girls [9,28], emotional loneliness was significantly related to CDV in girls, but not in boys. Additionally, effect sizes of these relationships were medium for girls and small for boys. Adolescents’ experiences of being rejected by peers or suffering offline dating violence victimization have been related to feelings of loneliness in boys and girls [43,62,63,64,65]. However, the relations between CDV and loneliness have not been previously analyzed. The gender differences in the correlations herein obtained in this study might indicate a more negative impact of CDV victimization for girls, probably linked with more social isolation in girls suffering CDV victimization. Nevertheless, due to the correlational nature of the data, they could also indicate that girls with more feelings of loneliness and a more negative assessment of their social network are more vulnerable to be cyber victimized in their romantic relationships. These girls might wish to overcome their loneliness through a romantic relationship, and this wish could cause them to be less aware of some cyber abuse behaviors or to be more tolerant to these behaviors to maintain the relationship and not be left without a partner. That is, loneliness might be a negative consequence, or also a risk factor, of CDV victimization in girls. Longitudinal studies are necessary to better understand how both variables influence one another. Regarding the correlations between CDV victimization and cyberbullying victimization (direct and indirect), the results confirmed a strong link among these variables in both boys and girls; the strength of the relationship was higher for girls (with an average large effect size) than for boys (with an average medium effect size). In a similar way to Borrajo et al. [21], the associations of CDV with cyberbullying were higher for cyber-aggression behaviors than for cyber-control behaviors. In both cyberbullying and CDV, perpetrators use electronic devices to attack, which makes access to the victim easy from anywhere and at any time [8]. A risky use of these devices by adolescents could increase their likelihood to be victimized by both peers and partners. Other common risk factors could also explain these links. Moreover, the correlations between these two forms of cyber-victimization suggest that situations of poly-victimization may well be usual for some adolescents, which coincides with previous studies that have highlighted the need to examine poly-victimization situations in adolescents more closely [42,55,56,66].

The analyses carried out to compare adolescents’ groups with different CDV victimization type and frequency gave some interesting results. In cyber-control victimization, significant differences appeared between frequently and never victimized adolescents (boys and girls) in depressive mood and indirect cyberbullying victimization, but significant differences were not found in these variables between never and occasionally victimized boys and girls. The effect sizes of the mean differences were from small to medium (with effect sizes ranging from 0.029 to 0.096). However, even small effect sizes can be relevant. As it has been pointed out in the literature [32], the effects among psychological variables could have cumulative effects over time. Thus, small effects at the level of single events could have potential consequences in the long run, when there is an accumulation across time and occasions for a given individual. Moreover, it is important to highlight that there were no differences in depressive mood and cyberbullying victimization between adolescents occasional and never cyber victimized by their partners. The experiences of occasional cyber-control victimization could be normalized in many adolescent couples, which they would not interpret as CDV and therefore would be less related to adolescents’ depressive mood and cyberbullying victimization. Similar to offline dating violence [31], adolescents’ occasional involvement in cyber-control behaviors could be more related to their lack of previous experience in romantic relationships [31] and their beliefs in myths of romantic love [11,29].

Another interesting finding was the gender differences observed in the relations between cyber-control victimization and loneliness. Adolescent boys suffering frequent, occasional or never cyber-controlled by their partners reported similar feelings of loneliness and similar assessment of their social network. In contrast, girls frequently victimized reported more feelings of loneliness and assessed their social network worse than those never victimized. Loneliness is defined as a negative emotional response to a discrepancy between the desired and achieved quality of one’s social network [65]. In adolescence, loneliness is closely related to the quality of relationships with peers [45], and bullying and cyberbullying victimization have been previously related to loneliness [48,63,67,68]. However, the experience of suffering frequent cyber-control by the partner showed links with loneliness only for girls. These results could have several explanations. One explanation could be that the experience of suffering frequent cyber-control by the partner has more negative consequences for girls. Adolescent girls may suffer high social isolation levels due to more cyber-control by their partners, who could monitor their relationships, force them to end friendships and reduce their social networks, which may increase their feelings of loneliness. A greater weight of the myths of romantic love in girls [11,29], with a larger internalization of some romantic myths, such as the association between control and love, the perception of love as suffering or the belief that it is necessary to have a romantic partner to be happy, could increase the risk of adolescent girls remaining in a romantic relationship in which they feel loneliness. Another possible explanation is that loneliness would be a higher risk factor to suffer cyber-control by a partner for adolescent girls. Possible previous experiences of peer rejection or feeling their friendships are poor quality could make girls more vulnerable to become involved in a romantic relationship in which they suffer frequent cyber-control. Future longitudinal studies should explore these possible explanations to better understand relationships between loneliness and cyber-control victimization in girls.

Regarding cyber-aggression victimization, not only was its prevalence lower than cyber-control, but also fewer differences were observed between adolescents frequently and occasionally cyber victimized. These two groups (frequently and occasionally cyber victimized) reported similar direct cyberbullying victimization levels, which suggests that direct cyberbullying might be related more to cyber-aggression than cyber-control. Both cyber-aggression by partners and direct cyberbullying by peers are direct attacks on victims in order to harm them, and the victims of these two forms of cyber violence could share common characteristics, such as a risky use of electronic devices. Walrave et al. [8] highlighted in their review that cyberbullying and CDV are interrelated, share similar risk factors and are often associated with similar problem behaviors. Thus, it would be worthwhile conducting more studies to analyze to what extent being cyber victimized in one interpersonal context (peers or partner) may increase the risk of also suffering cyber victimization in another context. Offline dating violence victimization and peer victimization are related [43], and these links can be extended to the virtual world. With respect to the psychosocial adjustment of cyber-victimized adolescents, one unexpected result was that no significant differences in depressed mood or loneliness in adolescent boys suffering never, occasionally and frequent cyber-aggression by their partners were observed. Conversely, the adolescent girls who frequently suffered cyber-aggression by their partners reported more depressive mood, more feelings of loneliness and assessed their social network worse than the girls never cyber victimized. Although the effect sizes of these mean differences were small (ranging from 0.030 to 0.052), these effects could be relevant for frequently victimized girls, as it has been pointed out [32]. The gender differences in psychosocial adjustment observed in this study could be related to differences in the severity and consequences of these behaviors for boys and girls. Cyber-aggression behaviors suffered by girls may be more serious, or this type of cyber-aggressions by partners has a stronger negative impact on them. Adolescent girls could suffer more severe cyber-aggressions (e.g., serious threats) than boys, or girls attach more importance to suffer serious self-image deterioration on social networks when humiliating and degrading intimate information, photos and images are widely spread. It is also possible that gender differences appear in the co-occurrence of online and offline dating violence victimization. More severe cyber-aggression behaviors or a greater co-occurrence of offline and online dating violence victimization in girls could explain the gender differences observed in psychosocial adjustment. Another variable that should be analyzed is the context in which the cyber-aggressions suffered by boys and girls occur and the possible gender differences in the use of ICTs in adolescent couples characterized by mutual aggressions. Adolescent girls might consider that online context is safer if they decide to apply violence to their partners in romantic relationships in which mutual aggression occurs. All these questions need to be analyzed in more depth in future studies.

This study has some limitations that must be considered. First, it is a cross-sectional study and it is, therefore, impossible to provide explanations about the causality and influence of one variable on another. Longitudinal studies are necessary to explore the causal relationships between these variables. Another limitation of this study is that it did not consider the severity of the suffered CDV behaviors. Although a distinction between cyber-control and cyber-aggression behaviors was made in this study, the severity of suffered cyber-aggressions (e.g., severity of threats or types of humiliating information and photos spread on social networks) was not taken into account. The severity of cyber aggressions suffered by adolescent boys and girls could differ. Moreover, the possible co-occurrence of offline and online dating violence victimization was not contemplated. Both the severity of cyber-aggressions and the co-occurrence of online/offline dating violence victimization could imply different consequences for adolescents’ psychosocial adjustment, and they should be considered in future studies. Another limitation of this study was the use of self-report measures, which may include some biases based on adolescents’ perceptions. These measure types are frequent in studies on victimization and aggressive behavior in adolescents. Nevertheless, it would be worth jointly including other sources of information, such as partners. The possible bi-directionality of CDV also needs to be considered. As mutual verbal-emotional aggressions are frequent in adolescent couples [69], it would be worthwhile measuring not only CDV victimization, but also CDV perpetration to better understand dating violence in adolescents, and how it is related to adolescents’ psychosocial adjustment. Furthermore, some variables related to adolescents’ use of electronic devices (e.g., how many hours connected a day, or the type of risky use with these devices) that could increase the risk of suffering CDV should also be analyzed. Finally, possible cultural characteristics that may affect teen dating violence should be also considered in future studies. In this regard, Viejo et al. [70] compared prevalence and characteristics of physical teen dating in the United Kingdom and Spain, and found similar prevalence and forms of physical dating violence in both countries. Nevertheless, research about cultural differences in offline and online teen dating violence is very scarce, and possible differences or similitudes in variables linked to this violence should be analyzed more in-depth.

## 5. Conclusions

The present study provides novel and interesting findings about cyber-control and cyber-aggression victimization prevalence in adolescent couples and how it relates to adolescents’ psychosocial adjustment and cyberbullying victimization. Regarding prevalence analyses, not only the type of CDV victimization (cyber-control and cyber-aggression), but also the frequency of CDV victimization suffered and the possible gender differences have been analyzed. Results showed a higher prevalence of cyber-control victimization than cyber-aggression victimization, mainly for experiences of occasional cyber-control victimization with a similar prevalence in boys and girls. The high prevalence of cyber-control behaviors (almost half the adolescents (44.1%) reported suffering at least one cyber-control behavior by their partners) suggests that some of these behaviors would not be perceived as cyber abuse by adolescents. Although the cyber-aggression victimization prevalence was lower (11.6%), these data highlight the need to develop prevention programs to prevent CDV among adolescents.

The results of this study also showed strong correlations between being a victim of cyberbullying and suffering CDV, which suggests the existence of possible common risk factors and the need to pay more attention to adolescents suffering poly-victimization situations. Studies on the relationships between cyberbullying and CDV victimization are still very scarce, and this study highlights close links between these two forms of cybervictimization among adolescents. Furthermore, it would be worth developing intervention programs aimed at jointly preventing cyberbullying and CDV. These intervention programs should help adolescents to be aware of the risky uses of their social networks, to know how to identify abusive behaviors (in relationships with both their peers and their partners) and to teach them to adequately cope with situations of cyber violence.

Regarding the psychosocial adjustment in those adolescents suffering CDV, the obtained results suggest a greater vulnerability of adolescents frequently victimized compared to those occasionally victimized, and the need to continue analyzing gender differences. In addition, the findings about the relations among CDV victimization, depressive mood and loneliness in adolescents showed interesting differences between boys and girls. Adolescent boys and girls suffering frequent cyber-control reported more depressive mood than those never victimized, but frequently victimized girls also indicated more emotional loneliness and assessed their social network worse. In cyber-aggression victimization, the differences between boys and girls were bigger. Frequently cyber-victimized adolescent girls, but not boys, reported more depressive mood, more emotional loneliness and assessed their social network worse. All these results indicate a worse psychosocial adjustment in adolescent girls suffering cyber-control and cyber-aggression by their partners. Future research should continue studying these gender differences in CDV for developing more effective intervention programs.

## Figures and Tables

**Table 1:** Distribution of adolescent boys and girls according to their cyber dating violence victimization experience.

Cyber-Control Victimization				
Gender	Total Sample N (%)	Never	Occasional	Frequent
**Boys**	262 (43.3%)	137 (22.7%)	80 (13.2%)	45 (7.5%)
**Girls**	342 (56.6%)	201 (33.3%)	104 (17.2%)	37 (6.1%)
**Total**	604 (100%)	338 (56.0%)	184 (30.5%)	82 (13.6%)
**Cyber-Aggression Victimization**				
**Gender**	**Total Sample N (%)**	**Never**	**Occasional**	**Frequent**
**Boys**	262 (43.3%)	219 (36.3%)	14 (2.3%)	29 (4.8%)
**Girls**	342 (56.6%)	315 (52.2%)	11 (1.8%)	16 (2.6%)
**Total**	604 (100%)	534 (88.4%)	25 (4.1%)	45 (7.5%)

**Table 2:** Means, standard deviations and correlations among variables (boys above the diagonal).

Variables	1	2	3	4	5	6	7
1. Emotional Loneliness		−0.24 **	0.32 **	0.42 **	0.32 **	0.07	0.09
2. Social Network Assessment	−0.68 **		−0.10	−0.11	−0.20 **	0.01	−0.15 *
3. Depressive Mood	0.46 **	−0.36 **		0.13 *	0.06	0.17 **	0.13 *
4. Direct CB-V	0.47 **	−0.32 **	0.38 **		0.65 **	0.17 **	0.20 **
5. Indirect CB-V	0.34 **	−0.22 **	0.23 **	0.73 **		0.29 **	0.41 **
6. Cyber-control–CDV-V	0.22 **	−0.25 **	0.23 **	0.35 **	0.29 **		0.63 **
7. Cyber-aggression–CDV-V	0.25 **	−0.27 **	0.15 **	0.42 **	0.50 **	0.61 **	
*M* Boys	1.79 *	3.06	2.05 *	1.36 *	1.15	1.34	1.12 *
*M* Girls	1.89 *	3.05	2.22 *	1.47 *	1.15	1.26	1.05 *
*SD* Boys	0.50	0.66	0.81	0.53	0.36	0.54	0.35
*SD* Girls	0.57	0.61	0.88	0.66	0.42	0.51	0.26
Cohen’s *d*	−0.19	0.01	−0.20	−0.18	0.01	0.15	0.20

Note: CB-V = cyberbullying victimization; CDV-V = cyber dating violence victimization; ** *p* < 0.01; * *p* < 0.05.

**Table 3:** Means (standard deviation) for loneliness, depressive mood and cyberbullying victimization in adolescent boys and girls with different cyber-control victimization experiences.

Boys					
**Variables**	**Never**	**Occasional**	**Frequent**	***p***	*η* ^2^
**Em. Loneliness**	1.75 (0.55)	1.79 (0.42)	1.88(0.46)	0.362	0.008
**Social Network Ass.**	3.04 (0.68)	3.14 (0.64)	2.99 (0.60)	0.404	0.007
**Depr. Mood**	1.99 (0.76) a	1.99 (0.80) a	2.36 (0.91) b	0.024	0.029
**Direct CB-V**	1.29 (0.50) a	1.33 (0.43) a	1.61 (0.70) b	0.002	0.045
**Indirect CB-V**	1.08 (0.25) a	1.13 (0.25) a	1.39 (0.61) b	<0.001	0.096
**Girls**					
**Variables**	**Never**	**Occasional**	**Frequent**	***p***	*η* ^2^
**Em. Loneliness**	1.82 (0.54) a	1.96 (0.60)	2.09 (0.63) b	0.014	0.025
**Social Network Ass.**	3.10 (0.60) b	3.07 (0.59) b	2.78 (0.70) a	0.014	0.025
**Depr. Mood**	2.13 (0.88) a	2.33 (0.83)	2.65 (0.92) b	0.002	0.035
**Direct CB-V**	1.34 (0.53) a	1.54 (0.71) b	1.96 (0.84) c	<0.001	0.085
**Indirect CB-V**	1.08 (0.23) a	1.18 (0.53)	1.43 (0.42) b	<0.001	0.065

Note: Em. Loneliness = emotional loneliness; Social Network Ass. = social network assessment; Depr. Mood = depressive mood; CB-V = cyberbullying *victimization*; a < b < c; *p* < 0.05.

**Table 4:** Means (standard deviation) for loneliness, depressive mood and cyberbullying victimization in adolescent boys and girls with different cyber-aggression victimization experiences.

Boys					
**Variables**	**Never**	**Occasional**	**Frequent**	***p***	*η* ^2^
**Em. Loneliness**	1.77 (0.50)	1.88 (0.46)	1.90 (0.47)	0.342	0.008
**Social Network Ass.**	3.11 (0.65)	2.89 (0.73)	2.81 (0.64)	0.041	0.024
**Depr. Mood**	2.01 (0.78)	2.34 (0.80)	2.31 (0.96)	0.065	0.021
**Direct CB-V**	1.30 (0.50) a	1.74 (0.62) b	1.64 (0.59) b	<0.001	0.070
**Indirect CB-V**	1.09 (0.28) a	1.21 (0.30) a	1.50 (0.60) b	<0.001	0.129
**Girls**					
**Variables**	**Never**	**Occasional**	**Frequent**	***p***	*η* ^2^
**Em. Loneliness**	1.87 (0.56) a	1.98 (0.47)	2.33 (0.80) b	0.006	0.030
**Social Network Ass.**	3.09 (0.59) b	3.03 (0.53) b	2.42 (0.81) a	<0.001	0.052
**Depr. Mood**	2.19 (0.86) a	2.78 (0.89)	2.84 (1.02) b	0.002	0.036
**Direct CB-V**	1.40 (0.58) a	2.08 (1.14) b	2.37 (1.13) b	<0.001	0.124
**Indirect CB-V**	1.10 (0.29) a	1.43 (0.76) b	1.84 (1.11) c	<0.001	0.155

Note: Em. Loneliness = emotional loneliness; Social Network Ass. = social network assessment; Depr. Mood = depressive mood; CB-V = cyberbullying victimization; a < b < c; *p* < 0.05.
